# Supplementary figures and images for: CRK2 controls cytoskeleton morphogenesis in Trypanosoma brucei by phosphorylating β-tubulin to regulate microtubule dynamics
Source: PLoS Pathog. 2023 Mar 22;19(3):e1011270. doi: 10.1371/journal.ppat.1011270 (PMC10069784; doi:10.1371/journal.ppat.1011270)

Figure S1

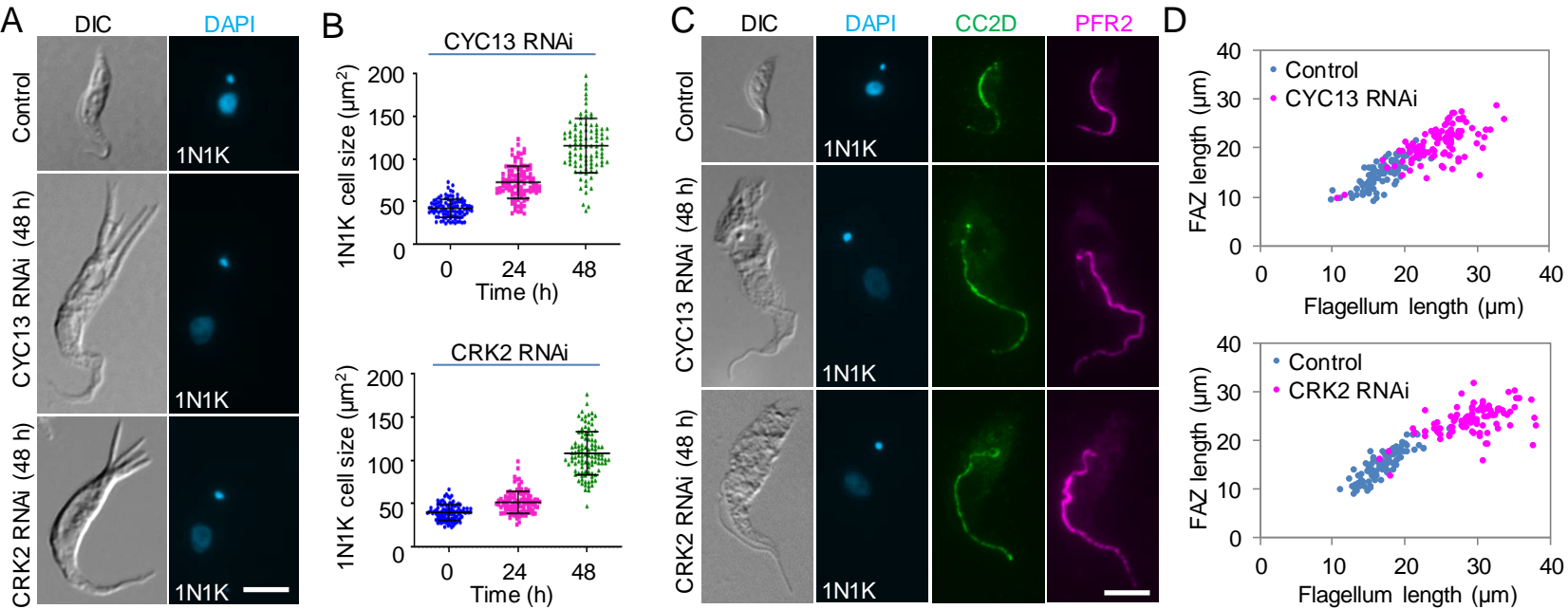

Supplement: S1 Fig — (A). Microscopic analysis of a non-induced control cells, CYC13 RNAi cells, and CRK2 RNAi cells. Scale bar: 5 μm. (B). Measurement of the cell size of non-induced control cells, CYC13 RNAi cells, and CRK2 RNAi cells. 100 1N1K cells for each time point were used for measurement. (C). Immunofluorescence microscopic analysis of the flagellum and its associated FAZ in non-induced control cells, CYC13 RNAi cells, and CRK2 RNAi cells. The flagellum was labeled with anti-PFR2 (clone L8C4) antibody, and the FAZ was labeled with anti-CC2D antibody. Scale bar: 5 μm. (D). Measurement of the length of the flagellum and the length of the FAZ in non-induced control cells, CYC13 RNAi cells, and CRK2 RNAi cells. RNAi was induced for 48 h. (PDF) [file ppat.1011270.s002.pdf]

Figure S2

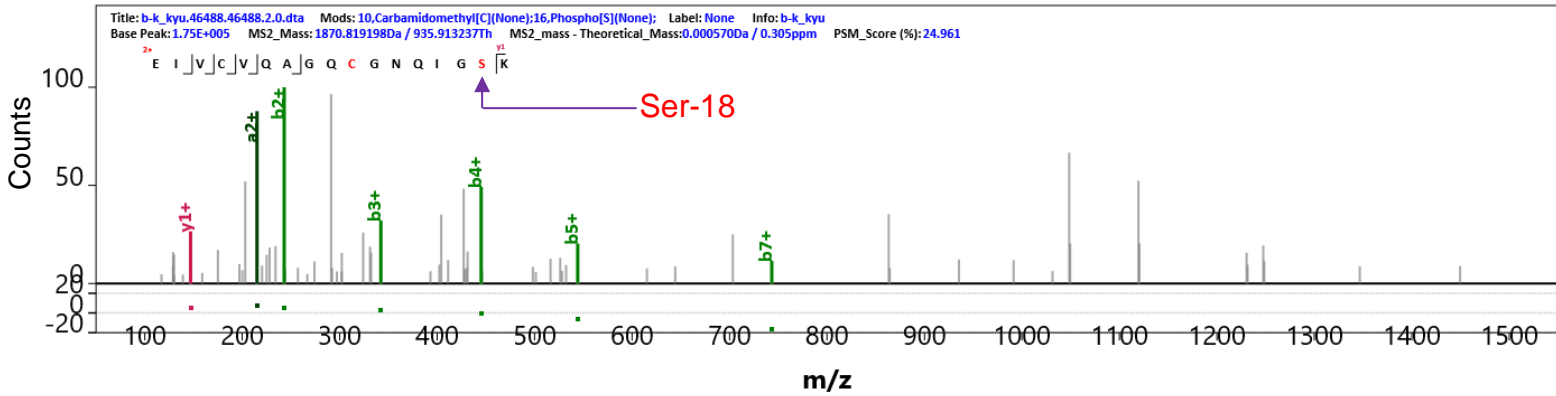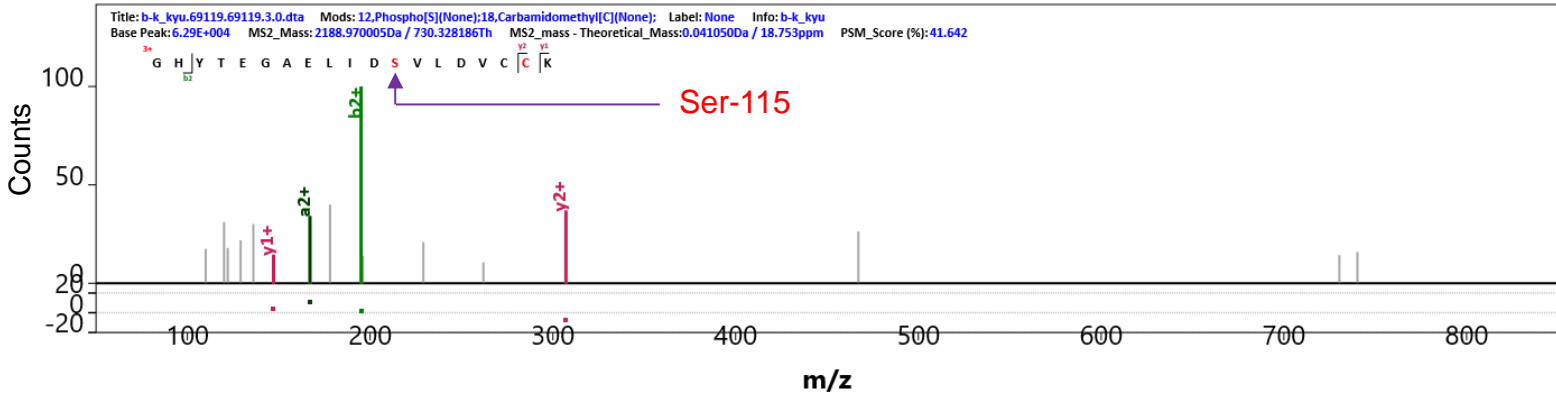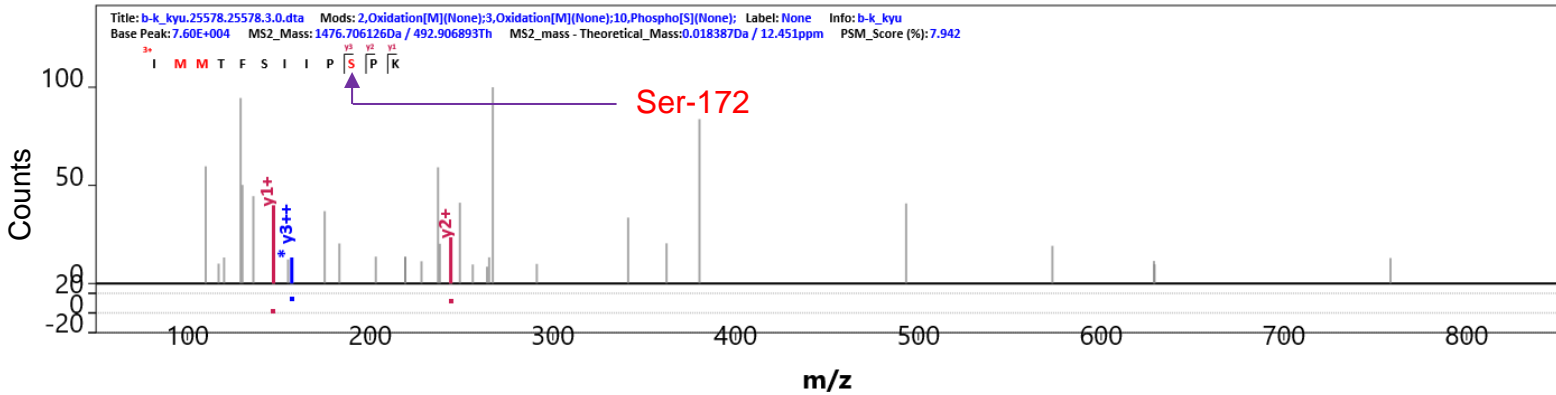

Figure S1 (continued)

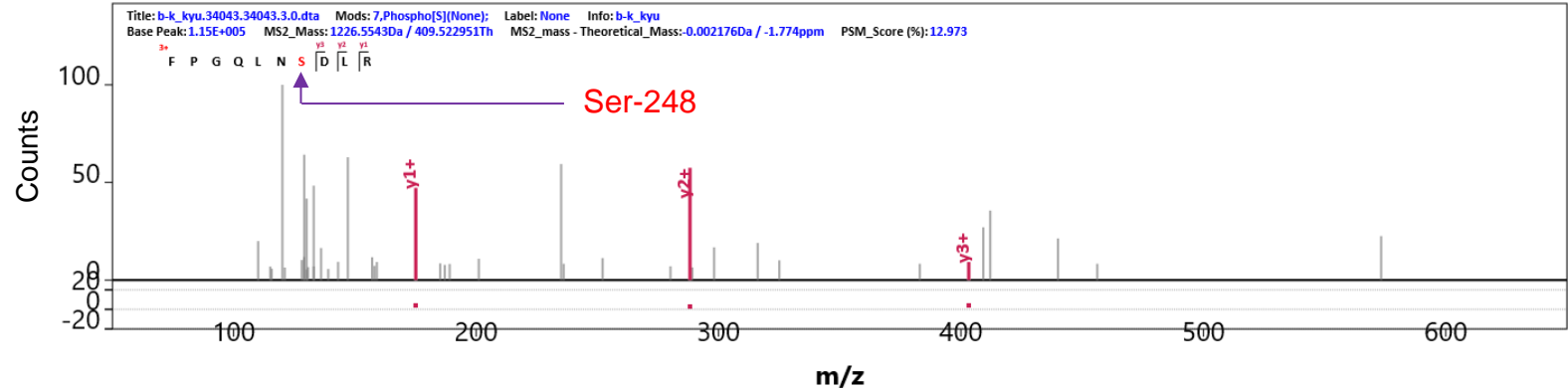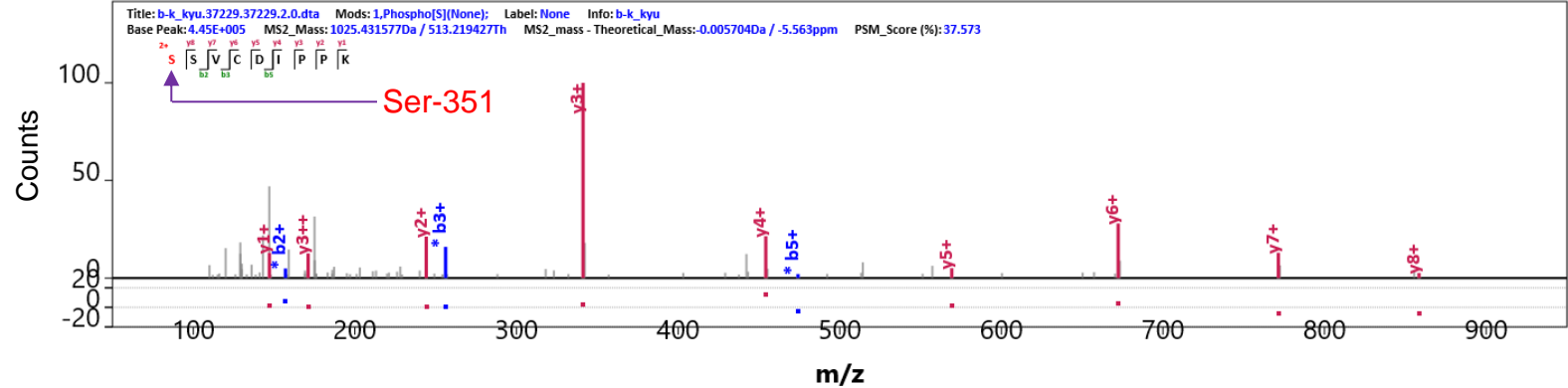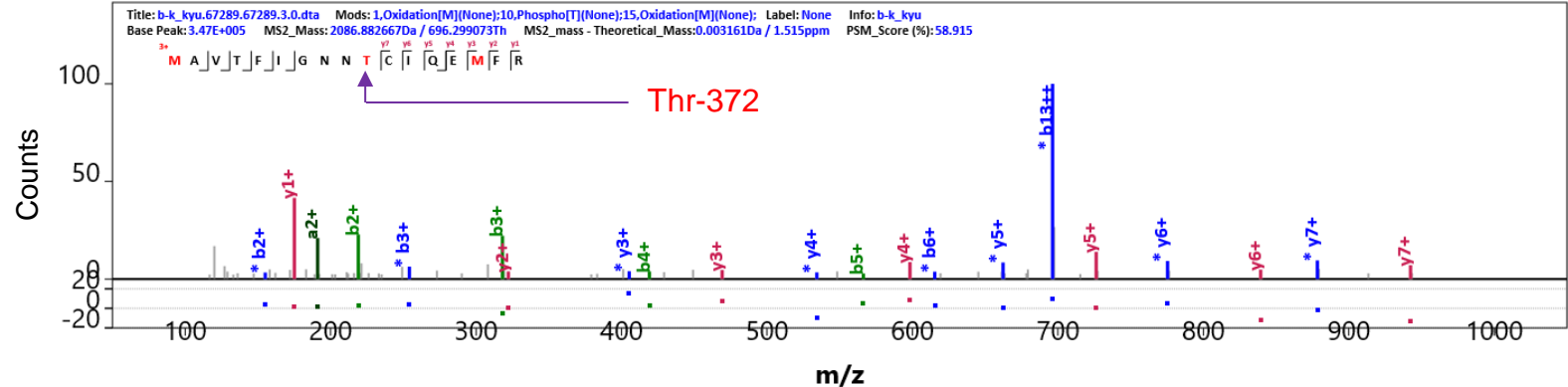

Supplement: S2 Fig — (PDF) [file ppat.1011270.s003.pdf]

Figure S3

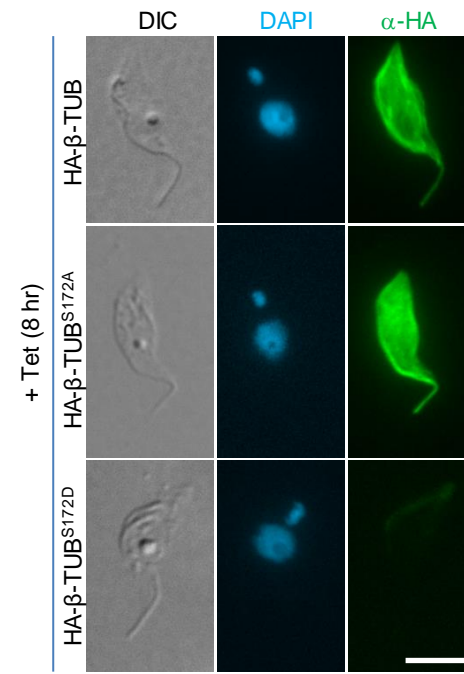

Supplement: S3 Fig — Shown are immunofluorescence microscopy images of HA-tagged β-tubulin and the S172A and S172D mutants after tetracycline induction for 8 hours. (PDF) [file ppat.1011270.s004.pdf]

Figure S4

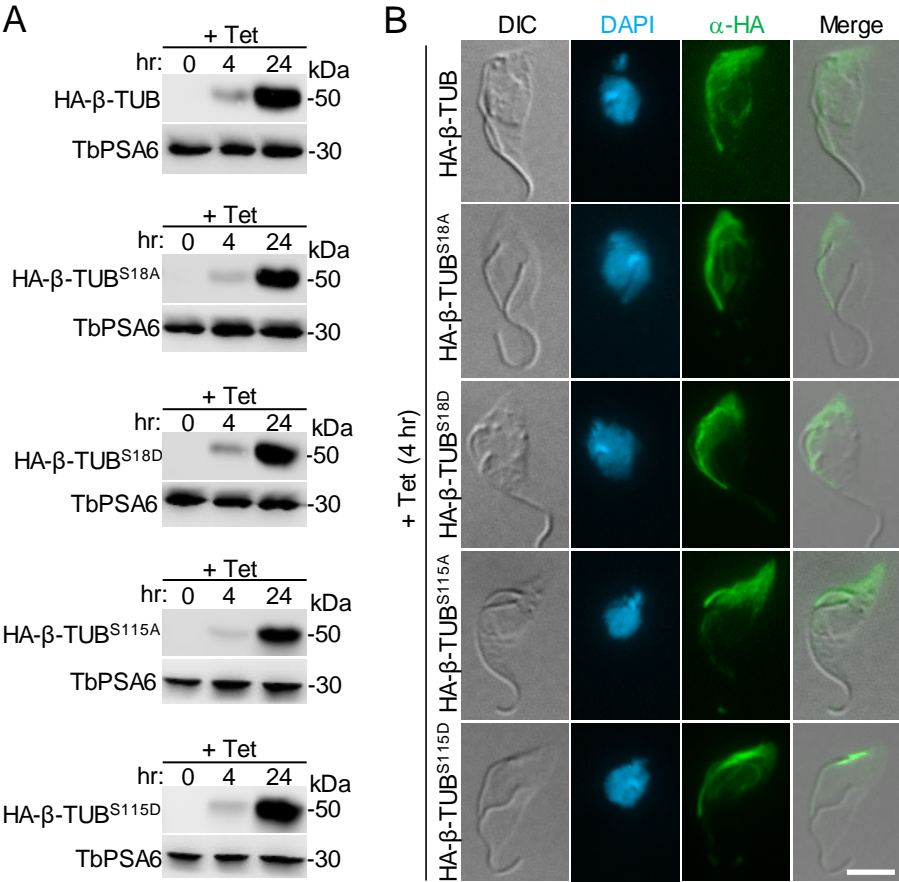

Supplement: S4 Fig — (A). Western blotting to detect the ectopically expressed control tagged β-tubulin, the S18A mutant, the S18D mutant, the S115A mutant, and the S115D mutant tagged with an N-terminal HA epitope. TbPSA6 served as a loading control. (B). Incorporation of control tagged β-tubulin and its mutants into the corset microtubules examined by immunofluorescence microscopy. Detergent-extracted cytoskeletons of T. brucei cells expressing HA-tagged β-tubulin or its mutants were immunostained with the FITC-conjugated anti-HA antibody. Scale bar: 5 μm. (PDF) [file ppat.1011270.s005.pdf]

Figure S5

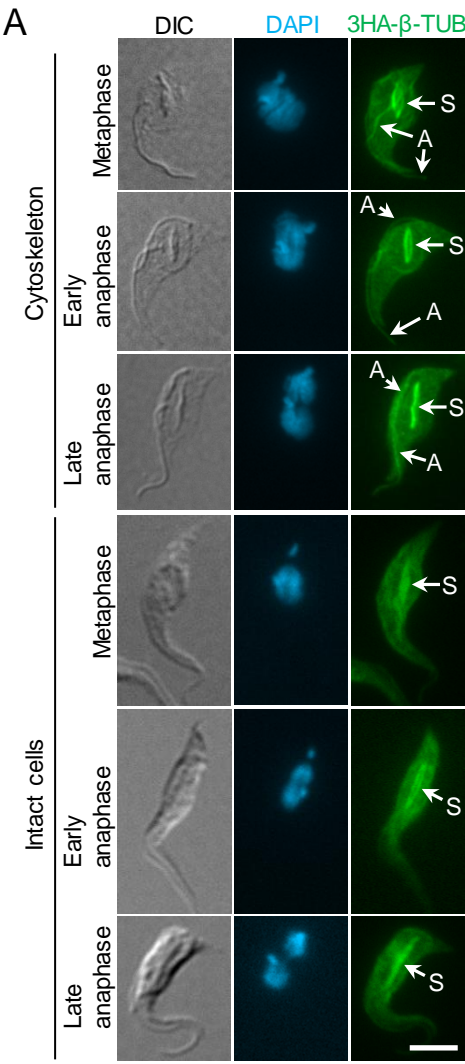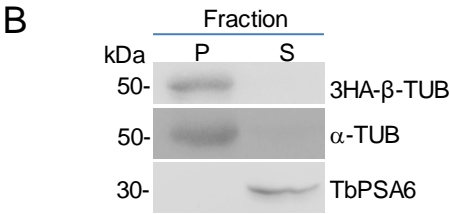

Supplement: S5 Fig — (A). Immunofluorescence microscopic analysis of 3HA-β-tubulin in intact cells and detergent-extracted cytoskeletons. A, axoneme; S, spindle. Scale bar: 5 μm. (B). Distribution of endogenous 3HA-β-tubulin in the cytosolic and cytoskeletal fractions of T. brucei cells. TbPSA6 served as the cytosol marker. α-tubulin served as the cytoskeleton marker. (PDF) [file ppat.1011270.s006.pdf]

Figure S6

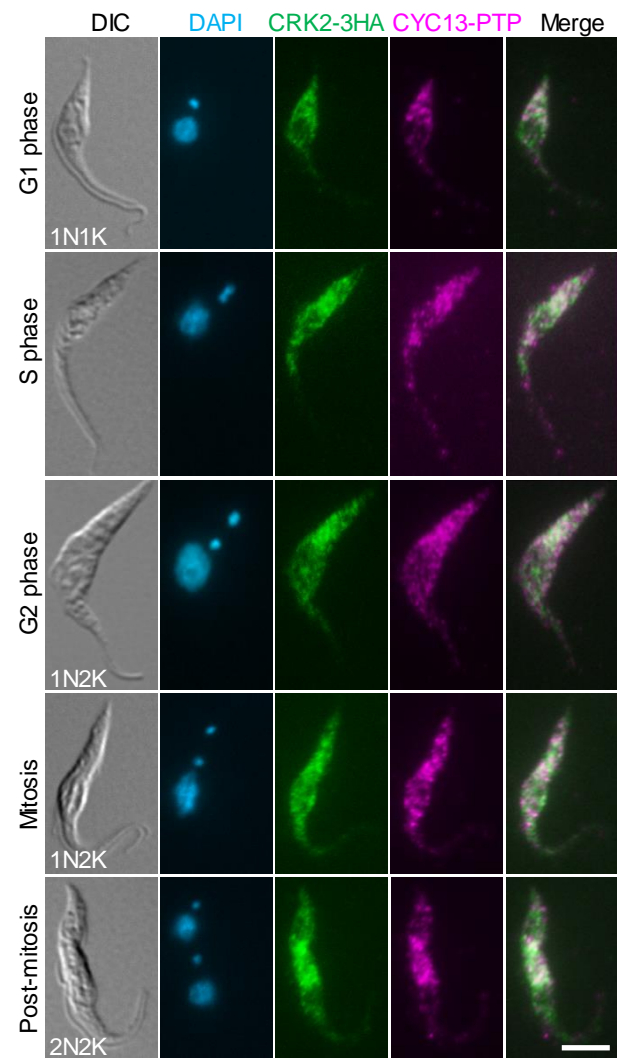

Supplement: S6 Fig — CRK2 was endogenously tagged with a triple HA epitope and CYC13 was endogenously tagged with a C-terminal PTP epitope. Cells were immunostained with the FITC-conjugated anti-HA monoclonal antibody and anti-Protein A polyclonal antibody, and counterstained with DAPI. Scale bar: 5 μm. (PDF) [file ppat.1011270.s007.pdf]
