# Supplementary material for: CRK2 controls cytoskeleton morphogenesis in Trypanosoma brucei by phosphorylating β-tubulin to regulate microtubule dynamics
Source: PLoS Pathog. 2023 Mar 22;19(3):e1011270. doi: 10.1371/journal.ppat.1011270 (PMC10069784; doi:10.1371/journal.ppat.1011270)
Supplement: Attachment — Submitted filename: Response to reviewers.docx [file ppat.1011270.s008.docx]

Response to reviewers’ comments

**MS ID#:** PPATHOGENS-D-22-01844

**MS title**: CRK2 controls cytoskeleton morphogenesis in *Trypanosoma brucei* by phosphorylating β-tubulin to regulate microtubule dynamics

**Authors**: Kyu Joon Lee, Qing Zhou, and Ziyin Li

We very much appreciate the most insightful comments and the very useful suggestions made by the three experts. We have revised the manuscript accordingly. The following is a point-by-point response detailing how we have dealt with the points raised by the reviewers.

**Part I – Summary**

Please use this section to discuss strengths/weaknesses of study, novelty/significance, general execution and scholarship.

**Reviewer #1**:

This manuscript is well written and there are some nice experiments testing the importance of CRK2 CYC13 in regulation of B-tubulin into the cytoskeleton. The first three results figures are very reasonable and the authors do show that knockdown of CYC13 and CRK2 by RNAi results in an increase of longer posterior ends, blunt ends and accumulation of tyrosinated tubulin indicative of new microtubules. The organisation of the extreme posterior end looks abnormal, but, surprisingly, XMAP215 is still localised into discrete foci, suggesting quite a level of microtubule minus end organisation even if there are multiple ends. The conclusion that CRK2-CYC13 might be regulating microtubule growth at the posterior end is reasonable, but regulation is quite hard to really test. Next, the authors tested if B-tubulin was a substrate of CRK2 and reported on six residues that reduced the level of phosphorylation. Two residues were extensively tested and found to result in a decrease in the ability of B-tubulin to incorporate into microtubules and the authors conclude that there is an increase in cellular levels of B-tubulin in the CRK2 and CYC13 RNAi cells. However, since the cells are larger overall, this might not be a correct conclusion. Whilst this is a nice piece of work extending their initial characterisation, it does lack impact for the target audience for PLOS pathogens.

We thank you for reviewing our manuscript and rating it as a nice piece of work. This reviewer argue that “since the cells are large overall, this might not be a correct conclusion”, referring to the increased levels of β-tubulin in CRK2 RNAi cells and CYC13 RNAi cells. We agree that the cells are larger and we are well aware of this when designing our experiments. We loaded equal amounts of proteins, not proteins from an equal number of cells, to the SDS-PAGE for western blotting (this was stated in the legend of Fig. 8B).

We respectfully disagree with the point that our work lacks impact for the target audience for PLOS Pathogens. PLOS Pathogens is a leading journal in the field, and it covers all aspects of pathogen biology, pathogen-host interaction, and pathogenesis. Readers of this journal are bacteriologists, virologists, mycologists, and parasitologists, including the scientists working on *Trypanosoma brucei*. *T. brucei* is an important parasite, and the cytoskeleton of this parasite is critical for cell division, cell morphogenesis, and cell motility, etc. We believe that our work fits very nicely with the scope of this journal.

**Reviewer #2**:

In this manuscript, Lee et al. revisit the phenotype of two knockdown trypanosome cell lines, namely of the kinases CRK2 and CYC13. Through careful investigation combining light and electron microscopy, they show that the posterior end of these cells elongates substantially, with a clear extension of microtubules, often resulting in spectacular posterior rend branching. In the second part of the manuscript, they search for a mechanistic explanation to this phenomenon. As a working hypothesis, they rely on the work of Didier Job’s group who showed that beta-tubulin phosphorylation by CDK1 inhibits its incorporation in microtubules (ref 22). They produce a set of convincing data that beta-tubulin can be a substrate of CRK2 in vitro and then proceed to analyse the impact of several phosphorylation sites in trypanosomes, selected by a combination of criteria (experiments presented here, phospho-proteomic data, evolutionary conservation). Phosphorylation of two of these residues turned out to increase tubulin turnover and the authors propose a nice model whereby absence of CRK2 would slow down beta-tubulin turnover, allowing further extension of microtubules, hence providing a molecular explanation to the excessively long posterior end of knockdown cells. The work is of high quality and should appeal to parasitologists but also cell biologists interested in cytoskeleton formation. However, it faces a technological challenge that is discussed below, with possible suggestions. There are also some major implications of this work that would deserve to be discussed in the manuscript.

We thank you for reviewing our manuscript, rating our work as of high quality, and providing valuable suggestions for us to improve the manuscript.

**Reviewer #3**:

There have recently been a number of papers documenting morphological phenotypes of the trypanosome cell posterior. While a range of different proteins have been implicated in generating this phenotype, the control mechanisms are unknown. In this study, Lee et al., make an intriguing mechanistic connection between this phenotype and the action of a mitotic kinase complex, which acts to regulate the incorporation of tubulin at the cell posterior.

The work builds on earlier studies which noted that depletion of the mitotic kinase CRK2 has an effect on the cell posterior end. The Li group recently demonstrated that CRK2 interacts with CYC13, and in this study they revisit the posterior end phenotype in light of this interaction. They show that depletion of either CRK2 or CYC13 produces the same phenotype, and elegantly show that the cell size changes can be attributed solely to an elongation of the cell posterior. A combination of fluorescence microscopy and electron microscopy is used to show that this posterior end elongation can be traced to increased deposition of newly-synthesised (i.e. still tyrosinated) tubulin, and that the microtubule plus ends are now extended in parallel instead of being gathered together to generate the normal tapered cell posterior.

They subsequently show that beta-tubulin can pull down CRK2 from cell lysates, and that CRK2 can phosphorylate specific sites on beta-tubulin in vitro. Mutagenesis of two of these sites to mimic phosphorylation results in lower levels of expressed protein and reduced incorporation into the cytoskeleton - effects that they show are likely due to a shorter half-life brought on by increased proteasomal degradation of the phosphomimic proteins. Lastly, they obtain evidence that when CRK2 or CYC13 are depleted, the cellular levels of beta-tubulin are increased.

This is a very attractive mechanism, and one that has been previously documented in mammalian cells using a similar set of approaches (Fourest-Lieuvin et al., 2006). What is intriguing is that the same mechanism is being used here for maintenance of cell morphology, and is apparently being coordinated by CRK2 instead of CDK1.

It is a nice, self-contained story that suggests lots of interesting follow-up questions. The data are of good quality throughout, rigorously quantified, and feature an attractive blend of techniques. It will undoubtedly be of great interest to the trypanosomatid community, and a valuable addition to the growing body of work on the mechanisms governing cytoplasmic microtubule organisation and cell posterior end morphology.

I have a couple of modifications which I think are essential, as well as a longer list of more minor changes and editorial alterations that the authors may wish to take on board, and which I think would strengthen the manuscript.

We are grateful to this reviewer for reviewing our manuscript, ranking our work highly, and providing valuable suggestions to improve the manuscript.

**Part II – Major Issues: Key Experiments Required for Acceptance**

Please use this section to detail the key new experiments or modifications of existing experiments that should be absolutely required to validate study conclusions.

**Reviewer #1:**

My main concern there is no evidence to show that cells are arrested in S-phase as outlined at the start of the results section and this is very important to show.

The roles of CRK2 and CYC13 on S-phase progression were previously characterized and published (Lee and Li (2021) The CRK2-CYC13 complex functions as an S-phase cyclin-dependent kinase to promote DNA replication in *Trypanosoma brucei*.  BMC Biology, 19: 29).

The timings chosen for analysis are a long time after initiation of RNAi and the phenotype of large posterior ends could be an accumulation over time leading the phenotypes observed rather than problems with the regulation of B-tubulin incorporation.

We carried out experiments from early time points of RNAi (8 hours and 16 hours after RNAi induction), and the results showed that RNAi of CRK2 or CYC13 both produced cells with an elongated posterior or an elongated and branched posterior (Fig. 1B). Please note that the RNAi cell lines generated with the 29-13 cell line have a doubling time of around 14-15 hours, not 8-9 hours as observed for the 427 cell line. Therefore, the posterior elongation phenotype emerged within the first cell cycle of the RNAi cell line. This is actually quite striking.

We all know that RNAi-mediated gene knockdown targets mRNA for degradation, and the emergence of phenotype depends on how fast the already-present target protein is degraded. Thus, it is typical for the phenotype to accumulate over time after RNAi induction because the target protein will be gradually depleted. In the case of posterior elongation after CRK2 RNAi or CYC13 RNAi, the posterior was slightly elongated after 8-16 hours and then massively elongated after 24-48 hours. This appears to be exactly the effect of microtubule extension, leading to gradual elongation of the posterior.

**Reviewer #2:**

The authors used an ectopic copy of N-terminally HA-tagged beta tubulin under the control of a tetracycline operator to express control or point mutations of the candidate phosphorylated residues (Figures 5 and 6). They call the HA-tagged tubulin “wild type” tubulin. However, this is somehow misleading because this protein seems to behave not exactly as wild-type. The fractionation experiments indicate that ~40% of HA-beta-tubulin is retained in the soluble pool, in contrast to wild-type beta-tubulin for which the large majority is incorporated into microtubules and the soluble pool is very low (see for example ref. 10). This is also an issue for Figure 7 where the authors used the same HA-tagged tubulin version to evaluate beta-tubulin abundance and to correlate it with microtubule extension. If close to half of the tagged protein were in the soluble pool as shown in control conditions, this weakens the argument. We suggest probing directly for endogenous beta-tubulin using the commercially available beta-tubulin specific antibody KMX-1 (Birkett et al 1985). Looking for alpha-tubulin would also be relevant (see below), for example with the TAT-1 antibody (Woods et al JCS89).

This is a great point that is very well taken. We agree that the HA-tagged β-tubulin expressed from an ectopic locus under a tetracycline-inducible promoter likely behaved differently from the wild-type, untagged β-tubulin. We have adopted the naming from this reviewer by calling the HA-tagged β-tubulin as “control tagged β-tubulin” to distinguish it from the wild-type β-tubulin.

These two reviewers suspect that only half of the endogenous 3HA-tagged β-tubulin was incorporated into microtubules in CRK2 RNAi cell line and CYC13 RNAi cell line, which might weaken our conclusion. To alleviate this concern, we have performed fractionation assay and found that endogenous 3HA-tagged β-tubulin was only detected in the pellet fraction (Fig. S5B). Moreover, endogenous 3HA-tagged β-tubulin can readily detect spindle, cytoskeleton, and the flagellar axoneme (Fig. S5A). It seems that HA-tagging of β-tubulin at the N-terminus from an endogenous locus does not evidently affect its function or incorporation into corset microtubules, spindle microtubules, and the flagellar axoneme. We suspect that the poor incorporation of ectopically expressed HA-β-tubulin into microtubules (Figs. 6 and 7) might be due to ectopic expression (although the amounts from ectopic expression can be low compared to the native tubulin, they are still excessive and are more than the amounts that can be incorporated into the cytoskeleton), rather than N-terminal epitope tagging. Anyway, we performed western blotting with the KMX-1 antibody, and we found that the level of β-tubulin detected by KMX-1 was similarly increased (Fig. 8B), which confirmed the results obtained with 3HA-β-tubulin.

We also performed western blotting with anti-α-tubulin antibody to detect the levels of α-tubulin in CRK2 RNAi cells and CYC13 RNAi cells. The results showed that the levels of α-tubulin were similarly increased (Fig. 8B). Given that β-tubulin levels were increased, and posterior microtubules extend massively, this result is expected. It appears that the levels of α-tubulin and β-tubulin are coordinated.

IFA images reveal that after 4 hours of expression, this HA-tagged beta tubulin is incorporated at the posterior part of the cytoskeleton but with a pronounced lateral bias visible on all images, including for the version with mutated residues. This might reflect specific aspects of beta-tubulin incorporation but this would be surprising since the profile looks different from tyrosinated alpha-tubulin that is being used as a classic marker of microtubule assembly (ref. 4). This is a matter of concern for the interpretation of the data as it questions whether the tagged version is indeed representative of the wild-type beta-tubulin.

Having said that, about 60% incorporation in microtubules is so far the best result for a tagged tubulin in trypanosomes. Tagging alpha-tubulin turned out to be even more challenging with most of the tagged protein remaining in the soluble fraction (Bastin et al 96, Sheriff et al 2014).

We think that calling this tagged beta-tubulin “control tagged tubulin” (or something equivalent) would be more appropriate. We suggest showing expression after longer induction periods (24 hours or more). If it leads to a homogenous staining of the cytoskeleton, this would be very reinsuring.

It is true that the dorsal edge of the posterior portion of the cell was intensively immunostained, but the posterior portion of the cell was also stained. The old images we chose have too strong signal at the dorsal edge, so it makes the signal at the posterior look weaker. We have replaced the old images with new ones that showed enriched immunofluorescence signal near the posterior end (Fig. 6D and Fig. 7D).

We also induced the expression of HA-tagged β-tubulin and its mutants for longer times, and we found after 8 hours of induction, the fluorescence signal can be detected throughout the cytoskeleton (Fig. S3). As stated above, it appears that N-terminal tagging of β-tubulin does not affect its incorporation into microtubules.

Second, there is no evidence that this is in fact an overexpression. There are roughly 40 copies of beta-tubulin genes in the genome and from the presented data we cannot infer if induced expression of the HA-tagged protein(s) is leading to a notable increase in overall beta-tubulin. We can suggest a control experiment with the commercially available beta-tubulin specific antibody KMX-1 (Birkett et al 1985) that works very well in trypanosomes. The presence of the tag should allow separation of the HA-protein from the endogenous beta-tubulin and will reveal the ratio between them on western blots.

We agree on this point. We did western blotting with KMX-1 on cells expressing ectopically expressed HA-β-tubulin, and it is indeed very hard to see any obvious overexpression. It is true that due to the presence of so many copies of β-tubulin genes in the genome, ectopic expression using the pLew100 vector apparently only produced a small amount of HA-β-tubulin. We have removed the wording “overexpression” from the entire manuscript, and instead we used “ectopic expression” throughout the manuscript.

Similarly, western blot analysis with antibodies recognizing PTM’s on beta-tubulin (glutamylation or detyrosination, see Casanova IJP15, van der laan et al. Cell reports 2009) would show that the tagged protein behaves reasonably normally.

We are unable to obtain these antibodies to do these experiments. However, based on the results obtained with endogenously HA-tagged β-tubulin, which showed that HA-β-tubulin was only detectable in the cytoskeletal pellet and can be detected in the spindle and the flagellum (Fig. S5). It seems that HA-tagging of β-tubulin does not affect its incorporation into microtubules.

Finally, we could not find a description on which tetracycline inducible system the authors used in this study. Please provide details.

We have added a paragraph in the Materials and Methods section to describe the tetracycline inducible system (lines 13-23, page 21).

Points for discussion.

The discussion section is a bit repetitive with the results, it could refocused to include three points that sound quite exciting to us.

The first is about alpha-tubulin. Beta-tubulin is not incorporated alone but always as a dimer with alpha-tubulin. This study focuses solemnly on beta-tubulin as the controller of integration. It is known that soluble fraction of both alpha and beta tubulin is low (ref 10). This begs the question how overabundance of beta-tubulin alone can lead to such a striking phenotype while the level of alpha-tubulin is presumably unchanged? Is phosphorylated tubulin degraded as a dimer or just beta-tubulin alone? This has implications on the abundance of tubulin-dimers during RNAi. Since microtubules elongate excessively in the knockdown experiments, these results suggest that either (1) it is the turn-over of beta-tubulin that decides that of alpha tubulin; (2) that both alpha- and beta- would be targets of CRK2 or (3) that alpha- and beta-tubulin regulate each other according to circumstances as they live as dimers. We are not asking for experimental evidence here as this would take too much time, but this point should be discussed.

Second thing, what controls the timing of expression/activity of CRK2? We imagine it is cell cycle-regulated, is there some evidence, maybe from previous work of the authors, that it peaks towards the end of the cell cycle? We know it is difficult to synchronise trypanosome cultures, but some localisation data might be available. If this is not the case, it is fine to elaborate in the discussion.

These two are great points! We indeed had thought about some of these points before. We were actually thinking that phosphorylation of β-tubulins might also affect the stability of α-tubulin, but we could not think of a way to test this possibility. We previously did the experiments to determine the subcellular localization of CRK2 and CYC13, and the data were included in supplemental Figure S6. We feel sorry that we did not try our best to discuss these points in the original manuscript. Your comments enlightened us, and we have tried our best to discuss them in the revised manuscript. We added one paragraph in the Discussion section (lines 20-27, page 18 and lines 1-17, page 19).

Third, in CRK2/CYC13 RNAi conditions, it is assumed that tubulin production is constant while integration is higher. The extension of microtubules however, would still be limited by the production of its main building blocks. Since soluble pool of tubulin is low, in order to facilitate the observed posterior elongation, the cell cycle length would probably need to be longer than usual. Unless lack of phosphorylation-mediated-degradation a) acts on the dimer and b) is substantial enough to deliver sufficient material to facilitate the observed elongation. Cell cycle disruption is observed when we consult the characterization of this RNAi cell line (ref. 15) where most cells stay in the 1K1N stage (CRK2 KD) and the growth is slowed down after the addition of tetracycline. One valid hypothesis is here presented: Decrease in phosphorylation leads to increase in tubulin-integration (and decrease of degradation) and therefore posterior elongation as well as cells failing to assemble a tapering end. However, it would be interesting to read a discussion on how a prolonged S-phase could contribute to posterior microtubule elongation. Is the cell cycle disrupted because of posterior elongation as a consequence of increased incorporation (and decreased degradation) or does the disrupted cell cycle facilitate excessive posterior elongation and branching by giving the cells more time to produce excess tubulin during S-phase and integrate it in the absence of phosphorylation?

This is also a great point. Because CRK2 plays roles in promoting DNA replication (S-phase progression) and restricting excess microtubule extension, it is reasonable to speculate that the two processes are dependent on each other. Especially for the cell cycle arrest, the prolonged S-phase progression definitely would give cells more time to extend the posterior microtubules. However, it is really hard to tell whether the S-phase defect is a direct consequence of posterior elongation. Conversely, it is also very hard to tell whether posterior elongation would cause cell cycle arrest, because so far all the reports about posterior elongation phenotype associate with cell cycle arrest at either G1 or S-phase. However, we don’t think the two cellular processes regulated by CRK2 are interdependent.

We have added a paragraph in the Discussion section to comment on this point (lines 18-26, page 19 to lines 1-7, page 20).

**Reviewer #3**:

1. P5 L12 I think an extra paragraph needs inserting here to provide more background context for the reader and to more fully acknowledge previous work in this area. The effect of cyclin depletion on the cell posterior was first noted in work done by the Mottram group (Hammarton et al., 2004 doi: 10.1074/jbc.M401276200), and the CRK2 phenotype was first observed by the Wang group (Tu & Wang, 2005; Tu et al., 2005 doi: 10.1091/mbc.e04-05-0368, 10.1242/jcs.02567). The de Graffenried group has also published two recent papers characterising proteins operating to maintain the morphology of the cell posterior (Hilton et al., 2018; Sinclair et al., 2021 - doi 10.1111/mmi.13986 and 10.1371/journal.ppat.1009588), and the Ersfeld group has (as already noted) characterised the effects of perturbing tubulin polyglutamylation - all this is essential background context for the reader and needs to come in the Introduction and not later on. P5 L24-26 (which are actually providing introductory information rather than results) can be absorbed into this paragraph. P13 L13-17 should also be relocated primarily to the Introduction. The original and interesting contribution of the work here is that it revisits the CRK2 phenotype in light of the more recent work from the Ersfeld and de Graffenried groups, and following the Li group's recent demonstration that CRK2 interacts with CYC13. It is of interest because it directly links the maintenance of the microtubule corset with cell cycle signalling complexes.

These are great points. We have added a paragraph to the Introduction section and included all the previous work on the regulation of posterior cytoskeleton morphogenesis (lines 16-27, page 5 and lines 1-3, page 6).

2. P9 L22+ I think it is essential that the authors here acknowledge (as, in fairness, they later do in the results and discussion) that phosphorylation of S172 in by a mitotic kinase was previously shown to affect the incorporation of beta-tubulin into microtubules in mammalian cells (Fourest-Lieuvin et al., 2006 - ref. 22). This is a plausible and attractive hypothesis to pursue, and it means that in their subsequent experiments they are demonstrating that a previously-observed mechanism also is operating in trypanosomes. What is interesting about their data is that it appears to be CRK2 and not CDK1 that is responsible for the phosphorylation event, and this is something they should comment on explicitly in their Discussion.

This is also a great point. We actually pointed out this before this paragraph (lines 18-20, page 10). However, we reiterated later again (lines 18-19, page 11).

**Part III – Minor Issues: Editorial and Data Presentation Modifications**

Please use this section for editorial suggestions as well as relatively minor modifications of existing data that would enhance clarity.

**Reviewer #1:**

Mainly with the first figure, but generally clearly written.

Page 6 line 3: It is not clear how the authors determined that the induced RNAi cells were arrested at S-phase and I could not find any supporting data.

As we have answered above, the roles of CRK2 and CYC13 on S-phase progression have been demonstrated and published previously (Lee and Li, BMC Biology, 2021).

Page 6 line 3-6: the description does not match figure 1B which is reported elongated or branched, but wording is describing posterior elongation.

The description of posterior branching follows the description of posterior elongation. We revised the counting of cells with an elongated posterior or an elongated and branched posterior. Because the branched posterior is also elongated, our previous counting included the cells with branched posterior in the category of cells with elongated posterior. In the revised counting, we have the category of cells with an elongated posterior and the category of cells with an elongated and branched posterior (Fig. 1B). We believe this new classification causes less confusion than the previous classification.

Page 6 line 7-8 you should add the N number of the cells measured.

The N number of the cells measured was always included in the Figure legends.

Page 6 Fig 1 measurements. This needs a cartoon image for readers to understand. Your measurements show that K to A is also significantly longer, so does that suggest that the anterior end is also longer?

The measurement data are now separated into Fig. 2. We added a cartoon image in Fig. 2A and Fig. 2C for readers to understand. The distance from K to A in 1N1K cells is indeed longer, but we think that this was due to the migration of the K towards the cell posterior. If we were to define the anterior length, we would suggest the distance from N to A, as the N (nucleus) is likely not moved during the cell cycle. In this case, the N-to-A distance was only slightly (not significantly) increased (Fig. 2B).

**Reviewer #2:**

The end of the introduction (p5 line 12) is very abrupt, we suggest to introduce a paragraph to describe the previous work on CRK2/CYC13 and to explain the goals of the study. It will also fluidify the beginning of the result section (bottom page 5).

We have added a few sentences in this paragraph to describe the previous work on CRK2-CYC13. We think this addition improved the fluidity and the rationale. Please see lines 4-10 on page 6.

Page 5, lane 4. For non-trypanosome people, please mention all copies of tubulins are identical (no sequence variants as observed in Plasmodium or Drosophila for example).

Information was added (lines 6-7, page 5).

Page 5, line 9. Please note that the impact of tubulin detyrosination in trypanosomes has been investigated recently (van der laan et al. Cell reports 19).

Information was added and reference was cited (line 11, page 5, reference 14).

Page 13, line 15. These experiments were done by RNAi gene knockdown, not by gene depletion (same comment page 14, line 15).

Corrected. Thanks.

Figure 1:

A cartoon showing the change of distance between cellular landmarks in uninduced vs. induced conditions could help the reader to interpret the data depicted a bit quicker.

A cartoon was added to Fig. 2A (formerly Fig. 1).

We apologise for having written such a long review, but the paper is very interesting and we hope our suggestions will be helpful.

Daniel Abbühl and Philippe Bastin

Thank you, Daniel and Philippe, for these very insightful comments and valuable suggestions. We think the manuscript is much improved after incorporating the suggestions and comments from you and the other two reviewers.

**Reviewer #3:**

Minor points: (in manuscript order)

P4 L4. I would mention also that different tubulin isoforms, as well as PTMs, contribute to functional diversity. It's ok to focus on PTMs from then on.

Information was added (lines 4-6, page 4). Thanks for this great point!

P4 L15 I would recommend citing Sinclair & de Graffenried 2021 (doi: 10.1016/j.pt.2019.07.008) either additionally to or instead of the 1999 Gull one - the 2021 review is more up-to-date and focuses specifically on the corset.

Yes, we agree. We have additionally cited the Sinclair paper (line 16, page 4, reference #4)

P4 L17 I would recommend citing Robinson & Gull, 1995 (doi: 10.1083/jcb.128.6.1163) here instead of the review article.

Changed (line 18, page 4, reference #5).

P4 L19 I would recommend additionally citing Wheeler et al., 2013 (doi:10.1111/mmi.12436) here.

Cited (line 20, page 4, reference #7).

P5 L2 Just as a comment - as far as I'm aware, the polarity of the quartet microtubules has not been experimentally demonstrated, but they are assumed to have their plus ends at the cell anterior tip.

This is a great point that we have missed. Thanks. We revised this statement to reflect this fact (lines 3-5, page 5).

P5 L7 It might be worth commenting on how similar the clusters of alpha and beta tubulin genes are, if this information is readily available. This could have important consequences for post-translational modification, especially if the phosphorylation sites are not universally conserved.

Based on the comment from reviewer #2 above, the sequences of these copies of tubulins are identical. This information has been included (lines 6-7, page 5).

P5 L13 Maybe the authors should specify explicitly that they recently identified CYC13 as being in a complex with CRK2? This provides a rationale for investigating both, and revisiting the depletion phenotype.

We have added a few sentences in this paragraph to describe the previous work on CRK2-CYC13 (lines 4-10, page 6). These additions improved the rationale and fluidity. Thanks.

P5 L27. This statement needs clarifying. They authors show that CYC13 depletion results in the generation of cells with elongated or branched posteriors. This is a new observation. The morphological phenotype is similar to that documented following CRK2 depletion, which was already known. They then show that they can recapitulate previous descriptions of this phenotype in their RNAi cells.

Actually, it was previous unknown that knockdown of CRK2 caused posterior elongation and branching. The work from Tu and Wang showed that the double knockdown of CRK1 and CRK2 caused posterior elongation (Tu and Wang, MBoC, 2005; Tu et al., JCS, 2006). However, RNAi of CRK2 alone performed by Tu and Wang showed no growth defects and no posterior elongation (Tu and Wang, JBC, 2004). We believe that their RNAi of CRK2 was inefficient, as we used the same CRK2 gene fragment and the same RNAi construct (pZJM) for RNAi and we found no phenotype as well. We then used a different CRK2 gene fragment and used the stem-loop RNAi construct, and the phenotype was very strong (Lee and Li, BMC Biology, 2021). We believe that the posterior elongation phenotype observed in CRK1-CRK2 double RNAi cells is attributed to RNAi of CRK1 alone, because we observed similar percentage of elongated cells in our CRK1 RNAi cells (Liu et al., Mol. Microbiol. 2013).

We observed posterior elongation and branching after RNAi of CRK2 or CYC13 when we obtained our RNAi cell lines, and in the images published in our initial work on CRK2-CYC13, these RNAi cells have an elongated posterior or an elongated and branched posterior (Lee and Li, BMC Biology, 2021). Our previous work was mainly focused on the roles of CRK2-CYC13 in regulating S-phase progression and their phosphorylation of DNA replication factor Mcm3. This current work is a follow-up to characterize the effects of their knockdowns on posterior morphogenesis.

P6 L3. The authors could maybe explicitly define what they mean by "elongation" (twofold increase in cell length?). This is important because there are no negative controls in Fig 1B, and they therefore need to explain why these are presumably scoring 0% according to their classification.

This is a great point that is very well taken. We added a few sentences in the revised manuscript to describe how we classify “elongated posterior”. (lines 24-27, page 6)

Basically, we measured the posterior in control and RNAi cells, and any RNAi cells with the posterior length longer than the longest posterior of control cells are classified as cells with an elongated posterior. We are fortunate that there appears to be little leaky RNAi in non-induced control cells, so we did not observe any control cells with an obviously elongated posterior.

P6 The analysis presented in Fig 1CDE is impressive and very helpful.

Thanks.

P7 L23 I would recommend also citing Kilmartin et al., 1982 here (characterisation of YL1/2).

Reference was cited (line 14, page 9, reference #29).

P7 L24. I think this could be clarified a bit. It's probably more accurate to say that the images show that the RNAi cells have increased amounts of tyrosinated tubulin at the elongated posterior ends. The data in Fig2 suggest (I think) that the number of microtubules present is roughly the same as in controls; "filled with" could be read as meaning that the number of microtubules increases.

Great point! We have revised this part accordingly (lines 16-17 and 20-21, page 9).

P8 L18. I think this statement could perhaps be made more precisely. What the authors have shown is that when either CRK2 or CYC13 are depleted, the posterior end of the cell becomes elongated and that this can be attributed to the deposition of tyrosinated (newly-synthesised) tubulin.

Another great point! We have revised accordingly (lines 9-11, page 10).

P9 L1-2 and P24 L1. I wouldn't call this an "in vitro" interaction as the input is cell lysate rather than purified tubulin. It would be more accurate to say that beta-tubulin can pull down CRK2 from cell lysates. Was any attempt made to do a reciprocal pulldown, or demonstrate the interaction using a different technique?

We have removed the wording “in vitro” here and in other places. No, we didn’t do reciprocal pull-down or test the interaction using other techniques. However, the in vitro kinase assay experiments used purified recombinant proteins of both CRK2 and β-tubulin (Fig. 5B). The phosphorylation of β-tubulin by CRK2 may also indicate interaction, and it is indeed “ in vitro”.

Kinase-substrate interaction is often transient; thus, it is challenging to test it. Our intention of doing GST pull-down was to make sure they interact so that we can perform in vitro kinase assay to identify phosphosites.

P9 L3. A little bit more detail on the mutagenesis would be helpful. Has this mutant been used before and what is the role of the residue that is mutated? It would also be useful if a bit more detail on the kinase assay was provided - how is the phosphorylated product detected?

We have added the information about the essential role of this lysine residue for kinase activity (required for phosphotransfer reaction) and cited a reference (lines 25-26, page 10, reference #32). This mutation for CRK2 has not been used before, but mutation of the equivalent lysine residue in other protein kinases is the first choice for studying any protein kinases. We previously mutated the equivalent lysine residue in TbAUK1 (Li and Wang, Eukaryotic Cell, 2006) and TbPLK (Yu et al, JCS, 2012) and both disrupted kinase activity. There are numerous studies that used the same lysine residue mutation to disrupt kinase activity.

We added more information about the PNBM used in kinase assay in the M&M section. PNBM alkylates thiophosphates to form thiophosphate ester epitopes, which can be recognized by anti-thiophosphate ester (anti-ThioP) antibody.

P10 L24 Maybe say "being actively degraded" instead of "an unstable protein"? That's probably more precise. Similarly in P11 L2 it might be better to say that steady-state levels of the mutant protein are increased when proteasome activity is inhibited.

We agree with these great points. We have changed them accordingly (lines 26- 27 on page 12; lines 4-5 on page 13).

P12 L22-23 Given that the immunofluorescence microscopy is being done on whole cells, and given too that Figures 5B, 6B showed a substantial cytoplasmic fraction of beta-tubulin, I don't think Figure 7A provides information on whether incorporation of beta-tubulin into the corset microtubules is being affected. The authors would need to examine extracted cytoskeleton preparations to be more sure of that.

We did immunofluorescence microscopy using extracted cytoskeletons. The images in Fig 8A (formerly Fig. 7A) have been replaced.

P13 L16 The authors may wish to reconsider linking the CCCH zinc finger phenotype with the one described here. While clearly related to the CRK2 phenotype and worth mentioning in that context, the posterior "nozzle" phenotype caused by overexpression of the CCCH zinc finger protein does not involve any disruption of the organisation/gathering of the plus tips of the microtubules and so branching or extreme dilation of the posterior end is not seen (as far as I'm aware). This suggests that the phenotype seen with CRK2 (and other proteins causing branching/dilation of the cell posterior) results not just from elongation of the microtubules but also a loss in the organisaiton of their plus ends. The authors might wish to briefly comment/speculate on this?

This is a great point. We have added a paragraph in the Discussion section to comment on this difference (lines 11-27, page 16 to lines 1-2, page 17).

P15 L14 The authors might want to speculate why the microtubule elongation phenotype is seen only for the corset microtubules and apparently not for axonemal microtubules?

This is a great point. We actually performed the experiments to measure the length of the flagellum and the FAZ, using anti-PFR2 and anti-CC2D as markers, and the results are now included in supplemental Fig. S1C, D. It appears that both the flagellum and the FAZ in 1N1K cells are elongated (Fig. S1C, D). These results were not included in our original submission, because we wanted to focus on posterior elongation and branching, which was so clearly demonstrated and easier to characterize. We are fully aware that the two antibodies do not stain the axoneme and MtQ, but the increase in the length of flagellum and FAZ suggests that the axoneme and the MtQ should be similarly elongated. We think that these results make sense because RNAi of CRK2 or CYC13 should alter microtubule dynamics in all the microtubules of trypanosome cells (not including spindle microtubules due to the arrest of cells at S-phase in these RNAi cells).

We now included these data, along with the data showing the cell size was also increased (Fig. S1A, B), and we also added a paragraph in the Results section (lines 26-27 on page 8 and lines 1-9 on page 9) and a paragraph in the Discussion section (lines 3-21, page 17).

P16 L1 Consideration of where the phosphorylation event occurs is an excellent discussion point, and one that would be enriched with some information on the localisation of CRK2, which is not touched on here. Have the authors carried out fractionation to test whether any of the CRK2 is cytoskeleton-associated? Fluorescence microscopy coupled to mild extraction might also be relevant and would go beyond published data. These should be noted as possible future experiments at least.

This is a great point. We have included the localization of CRK2 and CYC13 during different cell cycle stages in intact cells (Fig. S6). It appears that CRK2 and CYC13 were somewhat concentrated at the posterior portion during early cell cycle stages, with enrichment at the posterior cell tip during S-phase (Fig. S6). It appears that both CRK2 and CYC13 were not able to resist detergent treatment, so they were not detectable on detergent-extracted cytoskeleton (data not shown). We believe that CRK2-CYC13 is unlikely to associate with cytoskeleton and that CRK2 phosphorylates β-tubulin before it is incorporated into microtubules (although these points have not been experimentally confirmed).

P16 L16 I think it would be more accurate to say that they have shown that a previously-documented control mechanism that utilises phosphorylation of beta-tubulin to regulate tubulin incorporation is also operating in trypanosomes, and coordinated by CRK2 instead of CDK1. Why CRK2 is used instead of CDK1 might be a good discussion point?

This is a great point that is well taken. In fact, on page 18, lines 7-20 we commented on this aspect, and pointed out the control mechanism for microtubule dynamics through phosphorylation of β-tubulin is conserved in humans. However, we further demonstrated that phosphorylation of β-tubulin promotes its degradation, which was not investigated in the previous work.

In this summary paragraph, we meant to state that we discovered a control mechanism for cytoskeleton morphogenesis, which was not investigated in the previous study and was likely different in humans because of the phosphorylation by a mitotic CDK. Therefore, we do not think the control mechanism for cytoskeleton morphogenesis through phosphorylating β-tubulin is previously documented. Our understanding is that it was previously documented that phosphorylating β-tubulin inhibits microtubule dynamics (likely during mitosis), but it is unknown whether this altered microtubule dynamics affect cytoskeleton morphology was not addressed.

Regarding CRK2 is used instead of the mitotic CDK homolog CRK3 is used to phosphorylate β-tubulin, we think that we cannot be ruled out the possibility that CRK3 can phosphorylate β-tubulin during mitosis, as this has not been investigated. We added one sentence in the Discussion section to point this out (lines 8-10, page 18).

P16 L16+ Perhaps the authors might also want to speculate on whether there are implications for this control mechanism in the context of morphological changes that occur at different stages in the life cycle, for example the formation of mesocyclic cells, long epimastigotes, and so on?

This is a great point. We would like to refrain from speculating the implications on the life cycle form development based on the current work, mainly because RNAi of CRK2 and CYC13 caused not only posterior elongation but predominantly branching (Fig. 1B), whereas the formation of long epimastigote does not involve posterior branching. Also the re-positioning of the kinetoplast is also different from that in long epimastigote cells. We think that this control mechanism of microtubule dynamics by CRK2-CYC13 is likely unrelated to the mechanism used during life cycle form transition.

P16 L26 For all cell lines, the authors should indicate how many separate clones were used for the different experiments, in order to control for biological variability. If only one clone was used, it is essential to demonstrate that the level of clone-to-clone variation is negligible.

We always characterize two clonal RNAi cell lines, and the phenotypes from the two clonal RNAi cell lines were almost identical. This information is now included in the M&M section (lines 1-3, page 21).

P17 L5 The authors should probably explicitly mention that depletion of CRK2 and CYC13 protein by RNAi was demonstrated in their previous work, which explains why it is not shown here.

Added. Thanks.

P17 L12 Was integration of the tagging construct at the endogenous locus confirmed by PCR analysis of genomic DNA?

Endogenous tagging was confirmed by PCR and subsequent sequencing of the PCR product. This information is now included in the M&M section (lines 10-11, page 21)

P20 L6-10 The authors make extensive use of statistical significance tests, but state only that a t-test or a Chi-squared test was used. There are many different kinds of t-tests (two-sample t-test, Welch's t-test...) and it's not clear which they used. It's also unclear which test was applied when, which is important as the data are sometimes numerical and sometimes categorical, and the number of treatment categories varies. I would personally advocate removing all the significance tests from the figures (the results seem pretty clear-cut in most instances) but if the authors would prefer to keep them, then they need to provide more detail in the figure legends about what test was used and why.

We agree on this point, and we have removed the statistical analysis from all figures.

P24 L2 The % fractions of the Input and pellet fractions need to be indicated (at the very least in the figure legend), so that the reader can get a sense of how much is being brought down.

Added. Thanks.

The authors may wish to consider converting their red/green images into magenta/green ones - this has better contrast and is compatible with colour blindness (applies to immunuofluorescence, graphs, and schematics in Figures 1, 3, 4, 5, 6, 7).

All red color panels have been changed to magenta color.

Editorial points, minor comments: (in manuscript order)

Abstract & Summary

P2 L3 - delete "human". T. brucei is primarily a parasite of animals, not just humans.

Agreed and deleted the word “human”.

P2 L3/4 - I think it would be more accurate to say "contains" or "features" instead of "is composed of" - there is more to the trypanosome cytoskeleton than just the corset microtubules.

Changed.

P3 L1 perhaps rewrite as "...is a vector-borne parasite causing human and animal African trypanosomiasis..."? T.brucei is a parasite not just of humans.

Changed.

Introduction
P4 L4 Delete the comma after "transport".

Deleted.

P5 L4 Delete "the" ("Clusters of alternating...").

Deleted.

Results
P6 L6 Maybe "more than five branches" instead? Calling the branches "posteriors" would suggest that normal posterior organisation is being duplicated in each branch.

Great point! We have revised everywhere in the manuscript to describe them as “posterior branches”. This is more precisely describing the phenotype. (line 6, page 7; lines 20 and 25, page 9; lines 3 and 4, page 10).

P9 L9 Replace "assay" with "screen".

Replaced (line 5, page 11).

P10 L2 Maybe clarify that mutation to alanine and aspartate respectively are used for the two states? Not all readers will be familiar with this kind of approach.

Changed (line 27, page 11). This improved the precision.

P10 L9 Replace "more" with "higher".

Replaced (line 9, page 12).

P10 L10 Replace "less" with "lower".

Replaced (line 9, page 12).

Materials and Methods

P17 L16 Replace "transformed into" with "used to transform". Some more details on expression time, lysis method, and elution of bound protein would be informative.

Changed. And more details about expression, cell lysis and purification were included. The recombinant protein was not eluted, but the protein bound to beads were used to incubate with trypanosome cell lysate containing CRK2-3HA. This information is also included in the M&M section (lines 1-14, page 22).

P17 L19, P19 L13 Specify whether "NP-40" is Nonidet P-40 or Tergitol type NP-40. Replace "prepared by centrifugation" with "clarified by centrifugation".

NP-40 is Nonidet P-40 (line 8, page 22). The other phrase was changed accordingly (line 9, page 22).

P18 L6 could the authors briefly mention what the PNBM is doing? (for the benefit of general readers)

Information was included (lines 1-3, page 23). This improved the clarity.

P18 L17 Maybe provide a little bit more detail on the trypsinisation - how much enzyme was used, and for how long?

We have added the detailed information about trypsin digestion and mass spectrometry (lines 11-27, page 23).

P18 L27 Mention what counterion was used for setting the pH of the PIPES. NaOH?

Yes. It was NaOH (line 5, page 24).

P19 L11 Pham et al., 2019 should be cited with a number, and is not currently listed in the References section.

Added. Thanks.

Figures/Figure legends

P22 L28 "causes" not "caused".

Changed.

P23 L10 "impairs" not "impaired".

Changed.

P24 L7 Maybe add a label to Figure 4 panel C to indicate that the phosphosites above the schematic are those identified following the in vitro assay, and the ones below the schematic are those identified by Ubaniak et al.?

Changed as suggested. Please see Fig. 5C (formerly Fig. 4C).

P24 L10 "highlighted" not "outlined".

Changed.

P24 L25 Figure 5 - I think it would be more accurate to say that the histogram shows the relative amounts of each tubulin construct present in the pellet and supernatant fractions.

Agree on this point. We have changed it accordingly.

P25 L6 I think it would be clearer to say that the beta-tubulin constructs were normalised against the loading control (TbPSA6) and then expressed relative to the first time point (0 hr).

Changed accordingly. Thanks.

Fig 6B - the labels say T351 but these should be S351.

All corrected. Thanks for pointing this out.

Figure 6

P25 - The comments made on Figure 5 (above) also apply here.

Changed accordingly.

Fig. S1

P26 L Maybe "data" instead of "spectrums"?

Changed.

Brooke Morriswood

University of Würzburg

Thank you, Brooke, very much for your insightful comments and suggestions. We are very grateful, and have incorporated your suggestions into the revised manuscript. We thank you and the other reviewers for your highly valuable reviews, which greatly improved our manuscript.
