# Supplementary material for: CRK2 controls cytoskeleton morphogenesis in Trypanosoma brucei by phosphorylating β-tubulin to regulate microtubule dynamics
Source: PLoS Pathog. 2023 Mar 22;19(3):e1011270. doi: 10.1371/journal.ppat.1011270 (PMC10069784; doi:10.1371/journal.ppat.1011270)
Supplement: Attachment — Submitted filename: Response to reviewers.docx [file ppat.1011270.s009.docx]

Response to reviewers’ comments

**MS ID#:** PPATHOGENS-D-22-01844R1

**MS title**: CRK2 controls cytoskeleton morphogenesis in *Trypanosoma brucei* by phosphorylating β-tubulin to regulate microtubule dynamics

**Authors**: Kyu Joon Lee, Qing Zhou, and Ziyin Li

Thanks again to the reviewers for their careful review of our revised manuscript. We have revised the manuscript accordingly. The following is a point-by-point response detailing how we have dealt with the points raised by the reviewers.

Reviewer #2: One point for correction: after having read again the van der laan et al. paper (Cell reports 2019, reference 14) about the role of detyrosination of tubulin, we noticed that these authors also showed an impact on posterior elongation of the cytoskeleton. Since tubulin detyrosination is supposed to increase its stability, this result goes along the line proposed in the current manuscript and should be added in the discussion alongside the point about glutamylation (page 16, line 15).

Thanks. We have added this point to the revised manuscript (lines 19-22, page 16).

Similarly, the sentence “only the polyglutamylation of microtubules has been functionally characterized (17,18)” of the introduction page 5, line 12) should be corrected since functional analysis is also presented for tubulin detyrosination, with the extension of the posterior end (and also some mitotic defects) in reference 14.

We have added this information to the manuscript (lines 12-14, page 5).

Finally, we noticed a few typos:

Page 5, lines 24 and 26 “polyglutamylation” (not polyglutamination)

Corrected. Thanks.

Page 18, line 22 “might peak” (not might be peaked)

Changed.

Page 20, line 6 “CRK2” (not CRk2)

Corrected.

Page 20, line 14 underlying mechanism *is* unknown

Corrected. Thanks.

Page 21, line 21 “expression” (not expressing)

Corrected.

Reviewer #3: 

EDITORIAL POINTS:

Author summary
P3 L3 Africa not African.

Corrected. Thanks.

Results
P8 L27 Quantitative not quantitatively

Corrected. Thanks.

P14 L22 "causes increased levels of" not "causes increased the level of"

Corrected. Thanks.

There are a few very minor additional corrections that can be made to the text from a language perspective, but I will leave that to the copy editors.

We have gone through the manuscript carefully and changed a few other typos and grammatical errors.
